# CX3CR1 deficiency suppresses activation and neurotoxicity of microglia/macrophage in experimental ischemic stroke

**DOI:** 10.1186/1742-2094-11-26

**Published:** 2014-02-03

**Authors:** Zhiwei Tang, Yan Gan, Qiang Liu, Jun-Xiang Yin, Qingwei Liu, Jiong Shi, Fu-Dong Shi

**Affiliations:** 1 Department of Neurology and the BNI-ASU Center for Preclinical Imaging, Barrow Neurological Institute, St. Joseph’s Hospital and Medical Center, 350 West Thomas Road, Phoenix, Arizona 85013, USA; 2 Department of Neurology, Key Laboratory of Neurorepair and Regeneration, Tianjin and Ministry of Education and Tianjin Neurological Institute, Tianjin Medical University General Hospital, Tianjin 300052, China; 3 Department of Neurosurgery, the First Affiliated Hospital of Kunming Medical University, Kunming 650031, China

**Keywords:** CX3CR1^-/-^, Microglia, Macrophage, Ischemic stroke, Middle cerebral artery occlusion

## Abstract

**Background:**

Chemokine (C-X3-C motif) ligand 1 (CX3CL1)/ CX3C chemokine receptor 1 (CX3CR1) signaling is important in modulating the communication between neurons and resident microglia/migrated macrophages in the central nervous system (CNS). Although CX3CR1 deficiency is associated with an improved outcome following ischemic brain injury, the mechanism of this observation is largely unknown. The aim of this study was to investigate how CX3CR1 deficiency influences microglia/macrophage functions in the context of its protection following brain ischemia.

**Methods:**

Wild-type (WT) and CX3CR1-deficient (CX3CR1^-/-^) mice were subjected to transient middle cerebral artery occlusion (MCAO) and reperfusion. The ischemic brain damage was monitored by rodent high-field magnetic resonance imaging. Neurological deficit was assessed daily. Neuronal apoptotic death and reactive oxygen species (ROS) production were analyzed by immunostaining and live imaging. Activation/inflammatory response of microglia/macrophage were assessed using immunohistochemistry, flow cytometry, 5-bromo-2-deoxyuridine labeling, cytokine ELISA, and real-time PCR.

**Results:**

CX3CR1^-/-^ mice displayed significantly smaller infarcts and less severe neurological deficits compared to WT controls, following MCAO. In addition, CX3CR1^-/-^ MCAO mice displayed fewer apoptotic neurons and reduced ROS levels. Impaired CX3CR1 signaling abrogated the recruitment of monocyte-derived macrophages from the periphery, suppressed the proliferation of CNS microglia and infiltrated macrophage, facilitated the alternative activation (M2 state) of microglia/macrophages, and attenuated their ability to synthesize and release inflammatory cytokines.

**Conclusion:**

Our results suggest that inhibition of CX3CR1 signaling could function as a therapeutic modality in ischemic brain injury, by reducing recruitment of peripheral macrophages and expansion/activation of CNS microglia and macrophages, resulting in protection of neurological function.

## Background

Ischemic brain injury elicits an inflammatory response that involves activation of microglia and monocyte-derived macrophages in the central nervous system (CNS). Within the lesion, these cells adopt unique molecular phenotypes with either protective or detrimental effects on neuron survival [1]. Despite this basic understanding, the mechanisms controlling functional diversity in microglia/macrophages remain elusive but undoubtedly include differential signaling via chemokines, cytokines, and other cues present in the ischemic microenvironment.

The chemokine (C-X3-C motif) ligand 1 (CX3CL1)/ CX3C chemokine receptor 1 (CX3CR1) signaling pathway has been shown to play an important role in the maintenance of neural-immune communication in health and disease [2,3]. Fractalkine (CX3CL1), a membrane-bound chemokine, is expressed predominantly by neurons in the healthy CNS [4,5] while its receptor (CX3CR1) is highly expressed on resident brain microglia and on peripheral immune cell populations including macrophages [6,7]. CX3CR1 selectively modulates microglia activity in response to its ligand CX3CL1 [6]. CX3CL1/CX3CR1 also regulates recruitment of circulating leukocytes to sites of injury [8,9].

Under neuroinflammatory conditions, loss of signaling via CX3CR1 has been shown to have either protective [10,11], or detrimental effects [12], or no effects at all [13,14]. These conflicting observations indicate the complexity and disease-specific regulation of neuron-microglia communication via CX3CL1 and CX3CR1. In regards to acute CNS injury models (transient and permanent brain ischemia, spinal cord injury), the collective data suggest that the absence of CX3CR1 significantly reduces ischemic damage and inflammation [15-17].

Although the processes of microglia activation, cytokine production, and phagocytosis have been studied in several animal models of ischemia injury in CX3CR1-deficient (CX3CR1^-/-^) mice, it remains unclear as to how CX3CR1 deficiency alters the function of these cells, resulting in reduced damage during ischemic injury (highlighted by several controversial findings). Following induction of focal cerebral ischemia within CX3CR1^-/-^ mice, no difference was observed in the number of IL-1β-expressing microglia; rather, decreased leukocyte infiltration is involved in the development of smaller infarcts [15]. Conversely, in an animal model of spinal cord injury, modest but significantly more recruited monocytes (CD45^hi^) accumulate in the spinal cord of CX3CR1^-/-^ mice by 3 days post-injury [16]; however, a decrease in the number of CD11b^+^/Ly6C^lo^/iNOS^+^ macrophages after 7 days post-injury was associated with reduced neuropathology and enhanced functional recovery in these CX3CR1-deficient mice [16]. To date, little is known about the CX3CL1/CX3CR1 pathway in the context of microglia activation following brain ischemia.

In the current study, using a murine model of transient middle cerebral artery occlusion (MCAO), we tested the consequences of the absence of CX3CR1 on microglia/macrophage proliferation/recruitment and on their neurotoxicity after brain ischemia. We find that loss of CX3CR1 signaling attenuates the monocyte recruitment, the expansion of resident microglia and newly recruited monocytes, and reduced their inflammatory capacity after MCAO, contributing to neuroprotection in CX3CR1-deficient mice.

## Materials and methods

### Animals

Breeding pairs of CX3CR1^-/-^ mice were obtained from The Jackson Laboratory (Bar Harbor, ME, USA). Knock-outs were generated by replacing the second exon of the CX3CR1 gene with the enhanced green fluorescent protein (GFP) reporter gene, and backcrossed for more than 10 generations to C57BL/6 [13]. Cells under control of the endogenous CX3CR1 locus (that is, microglia, macrophages, dendritic cells, and so forth) in homozygote CX3CR1^-/-^ mice are labeled with GFP and also lack CX3CR1 receptor function. Wild-type (WT) C57BL/6 mice were purchased from Taconic (Oxnard, CA, USA). All animals were housed in pathogen-free conditions at the animal facilities of the Barrow Neurological Institute, Phoenix, AZ, USA. All experimental procedures were approved by the Institutional Animal Care and Use Committee of the Barrow Neurological Institute and performed according to the Revised Guide for the Care and Use of Laboratory Animals.

### Transient middle cerebral artery occlusion and reperfusion

Adult male mice (aged 10 to 14 weeks, weight 24 to 27 g) were exposed to transient (90 minutes) focal cerebral ischemia induced by occlusion of the right middle cerebral artery using an intraluminal filament method [18,19]. The production of an infarct was confirmed by 2,3,5-triphenyltetrazolium chloride (TTC) [20]. The methodology for MCAO and TTC staining is detailed in Additional file 1: Supplementary Methods.

### Neurological deficit assessment

Daily neurological deficit assessment was performed by investigators blinded to the control and MCAO groups as described previously [19]. Rating scale: 0 = no deficit, 1 = failure to extend left forepaw, 2 = decreased grip strength of left forepaw, 3 = circling to left by pulling the tail, and 4 = spontaneous circling.

### Cerebral blood flow, apparent diffusion coefficient values, and T2-weighted images by rodent m*agnetic resonance imaging*

Magnetic resonance imaging (MRI) was performed on a 7-T small animal MRI, 30-cm horizontal-bore magnet, and BioSpec Avance III spectrometer (Bruker Daltonics Inc., Fremont, CA, USA). In order to assess whether the ischemia and reperfusion were induced successfully, single slice cerebral blood flow (CBF) images were acquired before MCAO, 1 hour after MCAO and 24 hours after reperfusion, using a Continuous Arterial Spin Labeling sequence. Multiple Segments Echo Planer Imaging sequences were used to acquire apparent diffusion coefficient (ADC) values 30 minutes after MCAO to assess the damage volume. T2-weighted images were acquired 24 and 72 hours after MCAO to evaluate the development of ischemic lesions, using a Rapid Acquisition with Refocused Echoes sequence. Detailed methodologies for these imaging are available in Additional file 1: Supplementary Methods. MRI data were analyzed using the MED × 3.4.3 software package (Medical Numerics Inc., Germantown, MA, USA) on a LINUX workstation.

### Reactive oxygen species assessment *in vivo*

Reactive oxygen species (ROS) generated in the brain were assessed in live mice by using the Xenogen IVIS200 imager (Caliper Life Sciences, Alameda, CA, USA) [21]. Briefly, mice were intraperitoneally injected with 200 mg/kg Luminol (Invitrogen, Carlsbad, CA, USA). After 10 minutes, bioluminescence images were captured with exposure time of 3 minutes. A region of interest tool was used to measure the chemiluminescent intensity of the whole brain. Data were collected as photons per second per centimeter squared using the Living Image software (Caliper Life Sciences).

### Immunohistochemistry

Terminally anesthetized mice were perfused intracardially with saline followed by 4% paraformaldehyde. The fixed brains were embedded in paraffin and cut into serial 6 μm thick coronal slides. Immunohistochemistry was performed with antibodies against Iba-1 (Wako Chemicals USA Inc., Richmond, VA, USA) to identify macrophages and microglia; 4-hydroxy-2-nonenal (4-HNE; Abcam, Cambridge, MA, USA) and 8-hydroxy-2-deoxyguanosine (8-OHdG; Abcam) to identify lipid peroxidation and damaged DNA of oxidative impairment. Immunolabeling was detected by applying the peroxidase-antiperoxidase procedure with 3,3′-diaminobenzidine as a co-substrate. For double immunofluorescent staining, antibodies against NeuN (Millipore, Billerica, MA, USA) and cleaved Caspase-3 (Cell Signaling, Danvers, MA, USA) were used to identify apoptotic neurons. Respective negative controls that omit primary antibodies and positive controls were applied for each case. The positive cells were counted at 20× magnification in matched sections. Results are presented as Iba-1^+^, 4-HNE^+^, 8-OHdG^+^ or cleaved-Caspase-3^+^/NeuN^+^ cells per mm^2^ within areas measured from 20× images using Image J.1.34vi software (National Institutes of Health) [12].

### Enzyme-linked immunosorbent assay

Brain homogenates were prepared from WT and CX3CR1^-/-^ mice 72 hours after MCAO. Animals were anesthetized and the brains were removed and immediately frozen in liquid nitrogen. The ipsilateral or contralateral hemisphere was homogenized in RIPA buffer (10 μl/mg brain, Sigma, St. Louis, MO, USA). Protein concentration was measured with bicinchoninic acid Protein Assay kit (Pierce, Appleton, WI, USA). The total protein concentration was adjusted to 1 mg/ml protein extract. The concentrations of IL-1β, IL-6, and TNF-α in brain homogenates were quantified by enzyme-linked immunosorbent assay (ELISA) kits (BioLegend, San Diego, CA, USA) and converted into pg/mg protein extract.

### 5-bromo-2-deoxyuridine labeling

In order to visualize proliferating mononuclear cells in CNS, mice were injected intraperitoneally with 5-bromo-2-deoxyuridine (BrdU) (50 μg/g of mouse weight in saline, Sigma) immediately before the MCAO procedure and again 24, 48, and 60 hours after surgery. At 72 hours after surgery, mice were anesthetized with isoflurane and transcardially perfused with PBS. Brains were removed, and microglia and invading leukocyte isolation was performed according to the standardized protocol described below.

### Isolation of mononuclear cells from the central nervous system

The isolation of microglia and invading leukocytes are based on discontinuous percoll gradients [22-24]. Briefly, fresh brain tissues were removed from mice and cut into ~2 mm pieces and incubated in 10 mM Hepes/NaOH buffer (10 mM HEPES, 150 mM NaOH, 7 mM KCL, 1 mM MgCl_2_, 1 mM MgCl_2_, 0.36 mM CaCl_2_) containing 1 mg/ml collagenase (Sigma) for 1 hour at 37°C. The tissues were dispersed with a syringe, filtered through a 100-mm wire mesh, and centrifuged at 2,000 rpm for 5 minutes at 4°C. After centrifugation, cell pellets were resuspended in 15 ml 30% Percoll (Amersham Biosciences, Piscataway, NJ, USA), and centrifuged against 70% Percoll in a 50-ml tube for 15 minutes. The cell monolayer at the 30 to 70% Percoll interface was collected and washed once for further staining.

### Flow cytometry

#### ***Cell phenotype***

The number of microglia/macrophage, their proliferation properties and inflammatory cytokine secretion were analyzed by flow cytometry. Single cell suspensions prepared from brain tissues were stained with fluorescently labeled antibodies: APC-CD45, PerCP-Cy5.5-CD45, PE-Ly6G, BV421-Ly6G, PE-Cy7-CD11b, PerCP-Cy5.5-BrdU, PE-IL-1β, V450-IL-6, or APC-TNF-α at designed combination. All antibodies and the isotype controls were purchased from BD Biosciences, San Jose, CA, USA. The staining was performed according to the manufacturer’s instructions and the details are available in Additional file 1: Supplementary Methods. After staining, samples were analyzed using a FACSAria Ι flow cytometer (BD Biosciences). To avoid the interference of GFP expressed on CX3CR1^-/-^ microglia/macrophage, fluors such as FITC and Alexafluor 488 that excite in the same spectrum at the same filter sets as GFP were excluded and the proper compensation was performed in flow cytometry. Subsequent data analyses were completed using FACSDiva software or FCS Express 4 software (BD Biosciences).

#### ***Cell sorting***

Microglia/macrophages were enriched from the single-cell suspension of the ischemic hemisphere of MCAO mice using CD11b MicroBeads (Miltenyi Biotec, Auburn, CA, USA), and then sorted via APC-45/PE-Cy7-CD11b/BV421-Ly6G makers from the remaining cells by FACSAria I using Diva software (BD Biosciences). Purity of microglia and macrophages obtained by this approach reached 99%.

### Quantitative real-time polymerase chain reaction analysis

Total RNA was extracted from the sorted microglia/macrophages using the RNeasy Micro Kit (Qiagen, Germantown, MD, USA) according to the manufacturer’s instructions; 1 μg was used to synthesize the first strand of cDNA using the Superscript First-Strand Synthesis System for real-time PCR (Invitrogen). PCR was performed on the Opticon 2 Real-Time PCR Detection System (Bio-Rad, Hercules, CA, USA) using corresponding primers (Table 1) and iQ^TM^ SYBR Green Supermix (Bio-Rad). Cycle conditions included heating for 5 minutes at 95°C, followed by 40 cycles of 30 seconds at 95°C, 30 seconds at 60°C, and 60 seconds at 72°C. A melt curve analysis was performed to ensure specific amplification. For each target gene, relative levels of expression were normalized against housekeeping gene GAPDH of the same sample. The relative expression levels of the mRNAs were then reported as fold changes versus sham controls.

**Table 1:** Primer sets for murine genes

**Gene**	**Encoded protein**	**Primer**
GAPDH	Glyceraldehyde-3-phosphate dehydrogenase	Forward: 5′- TGATGACATCAAGAAGGTGGTGAAG -3′
		Reverse 5′- TCCTTGGAGGCCATGTAGGCCAT-3′
**M1**		
iNOS	Nitric oxide synthase, inducible (iNOS)	Forward: 5′-CCCTTCAATGGTTGGTACATGG-3′
		Reverse: 5′-ACATTGATCTCCGTGACAGCC-3′
**M2**		
Mrc1	Mannose receptor, C type 1 (MRC1), Macrophage mannose receptor 1 (MMR), CD206	Forward: 5′-TCTTTGCCTTTCCCAGTCTCC-3′
		Reverse: 5′-TGACACCCAGCGGAATTTC-3′
Ym1	T-lymphocyte-derived eosinophil chemotactic factor (FCF-L)	Forward: 5′-GGGCATACCTTTATCCTGAG-3′
		Reverse: 5′-CCACTGAAGTCATCCATGTC-3′

### Statistical analysis

Results are presented as the means ± SEM. Statistical differences between two groups were evaluated by the two-tailed unpaired Student’s *t*-test. Multiple comparisons were performed with two-way analysis of variance accompanied by Bonferroni *post-hoc* test. Values of *P* < 0.05 were considered significant.

## Results

### Reduction of infarct volume and neurological deficit by CX3CR1 deficiency after middle cerebral artery occlusion

To ensure the success of the MCAO model, CBF of right middle cerebral artery territory was examined at 24 hours before MCAO, and 1 hour and 24 hours following MCAO using high-field MRI. Animals wherein ischemia and reperfusion were induced successfully were selected for further analysis (Additional file 1: Figure S1A). No significant difference in CBF at baseline (WT, 171.3 ± 9.5 vs CX3CR1^-/-^, 174.2 ± 10.1 ml/100 g/min, *P* > 0.05), ischemia (WT, 33.5 ± 14.3 vs CX3CR1^-/-^, 31.2 ± 13.1 ml/100 g/min, *P* > 0.05) and reperfusion (WT, 160.1 ± 15.1 vs CX3CR1^-/-^, 155 ± 12.3 ml/100 g/min, *P* > 0.05) was observed between WT and CX3CR1^-/-^ mice. Furthermore, ADC (measuring the magnitude of diffusion of water molecules within cerebral tissue) was acquired 30 minutes after MCAO to assess the damage volume of ipsilateral hemisphere (Additional file 1: Figure S1B, low value on the left). No significant differences in damage volume were observed between CX3CR1^-/-^ and WT mice (WT, 122.2 ± 8.3 mm^3^; CX3CR1^-/-^, 119.1 ± 6.7 mm^3^) (Additional file 1: Figure S1C).

**Figure 1:**
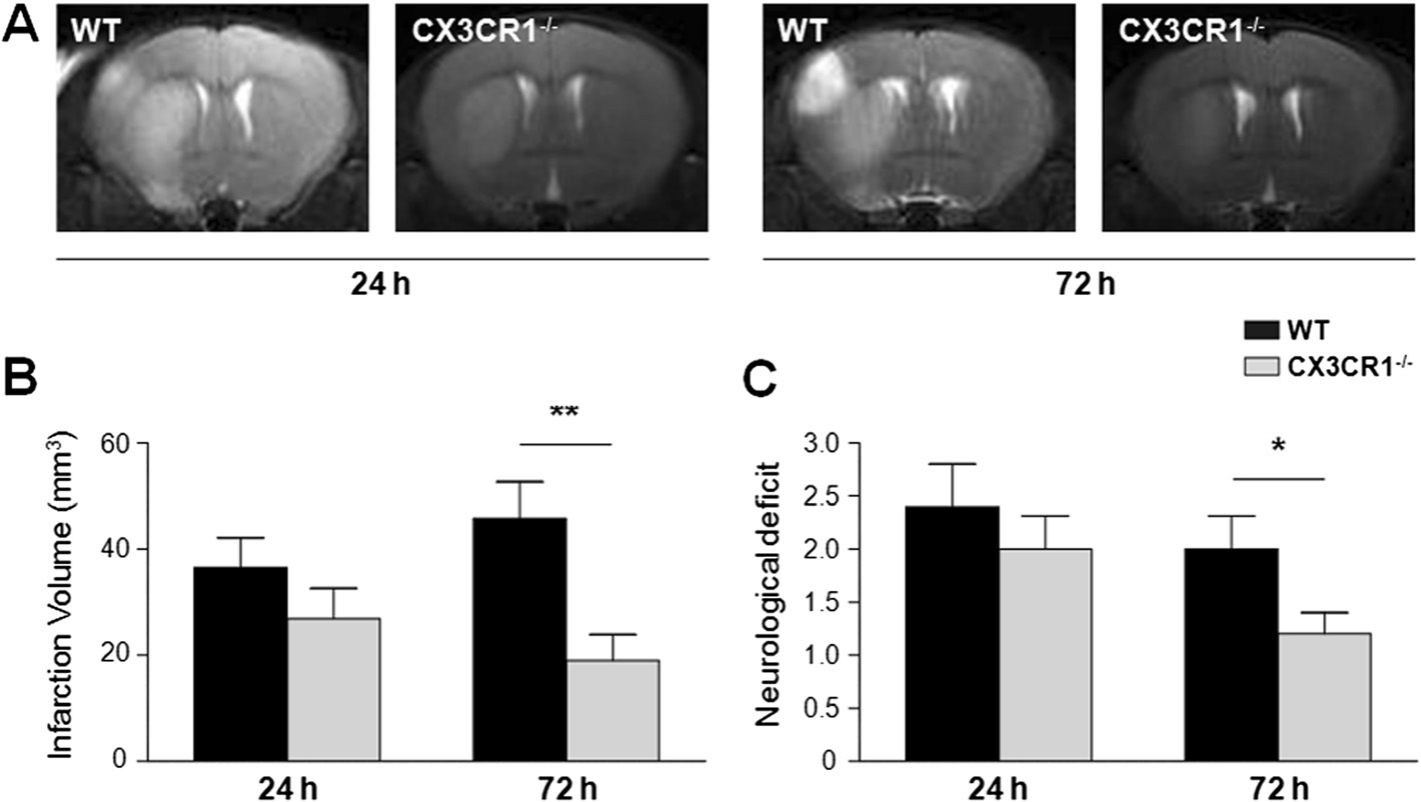
**CX3CR1 deficiency attenuates infarct volume and neurological deficit after middle cerebral artery occlusion. (A)** Infarct volume was assessed by T2-weighted images at 24 and 72 hours post-ischemia. **(B)** Quantification of T2 images shows that the infarct volume was attenuated by CX3CR1 deficiency 72 hours after middle cerebral artery occlusion (MCAO). *P* < 0.0001 for genotype, *P* = 0.0194 for time point, and *P* = 0.0011 for interaction by two-way analysis of variance. **P < 0.01 by Bonferroni *post-hoc* tests. **(C)** Clinical assessment demonstrated that CX3CR1^-/-^ mice have better neurological deficit scores than wild-type (WT) mice 72 hours after MCAO. *P* = 0.0049 for genotype, *P* = 0.0262 for time point, and *P* = 0.0062 for interaction by two-way analysis of variance. *P < 0.05 by Bonferroni *post-hoc* tests. *n* = 6 per group.

To assess the infarct volume, T2-weighted images were acquired at 24 and 72 hours following MCAO. The infarct volume in WT mice was 36.5 ± 5.7 mm^3^ at 24 hours and increased to 45.8 ± 6.8 mm^3^ at 72 hours. Within CX3CR1^-/-^ mice, infarct volume was 26.9 ± 5.7 mm^3^ at 24 hours, and a modest but significant decrease (to 19.0 ± 4.9 mm^3^) was observed at 72 hours (Figure 1A). Although there was no significant difference in infarct volume at 24 hours between the two groups, CX3CR1^-/-^ mice showed markedly smaller infarct volume at 72 hours relative to WT mice (*P* < 0.01, Figure 1B). The infarct observed in MRI scan was confirmed by TTC staining (Additional file 1: Figure S1D).

To further assess the differential response to MCAO in CX3CR1^-/-^ and WT mice, the neurological deficit was assessed daily following MCAO. WT mice had an average clinical score of 2.4 ± 0.4 at 24 hours and 2.0 ± 0.3 at 72 hours, while CX3CR1^-/-^ mice had clinical scores of 2.0 ± 0.3 at 24 hours and 1.2 ± 0.2 at 72 hours indicative of the beginning of recovery at 72 hours in CX3CR1^-/-^ mice but not in WT mice (Figure 1C).

### CX3CR1 deficiency attenuates neuronal apoptosis after middle cerebral artery occlusion

**Figure 2:**
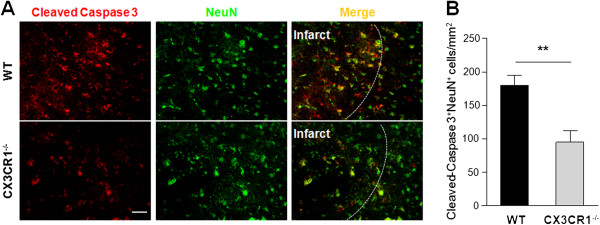
**CX3CR1 deficiency attenuates neuronal apoptosis after middle cerebral artery occlusion. (A)** Double staining with cleaved Caspase 3 (red) and NeuN (green) antibodies in peri-infarct area of wild-type (WT) and CX3CR1^-/-^ mice 72 hours after middle cerebral artery occlusion. **(B)** Quantification of Cleaved-Caspase 3/NeuN positive cells. CX3CR1^-/-^ mice have fewer Cleaved-Caspase 3^+^NeuN^+^ cells in the peri-infarct zone. **P < 0.01 by Student’s *t*-test. *n* = 4 per group. Scale bars = 50 μm.

Double staining with NeuN (neuron marker) and cleaved Caspase-3 (apoptotic marker) was performed to investigate whether CX3CR1-dependent differences observed in the size of ischemic damage at 72 hours following MCAO were associated with differential neuronal apoptosis in peri-infarct areas. More cleaved Caspase-3 positive cells were observed in WT mice compared to CX3CR1^-/-^ mice and co-localized with NeuN in most apoptotic cells (Figure 2A). The number of cleaved Caspase-3 positive neurons was 179.3 ± 15.6/mm^2^ in WT mice and 95.1 ± 16.9/mm^2^ in CX3CR1^-/-^ mice 72 hours after MCAO (*P* < 0.01, Figure 2B). To reveal the phenotype of the other apoptotic cells, double staining of cleaved Caspase-3 with Iba-1 or GFAP markers, respectively, were employed. These experiments revealed cleaved Caspase-3 positive staining within some microglia and astrocytes (data not shown).

### Fewer microglia and macrophages in ipsilateral hemisphere of CX3CR1^-/-^ mice after middle cerebral artery occlusion

**Figure 3:**
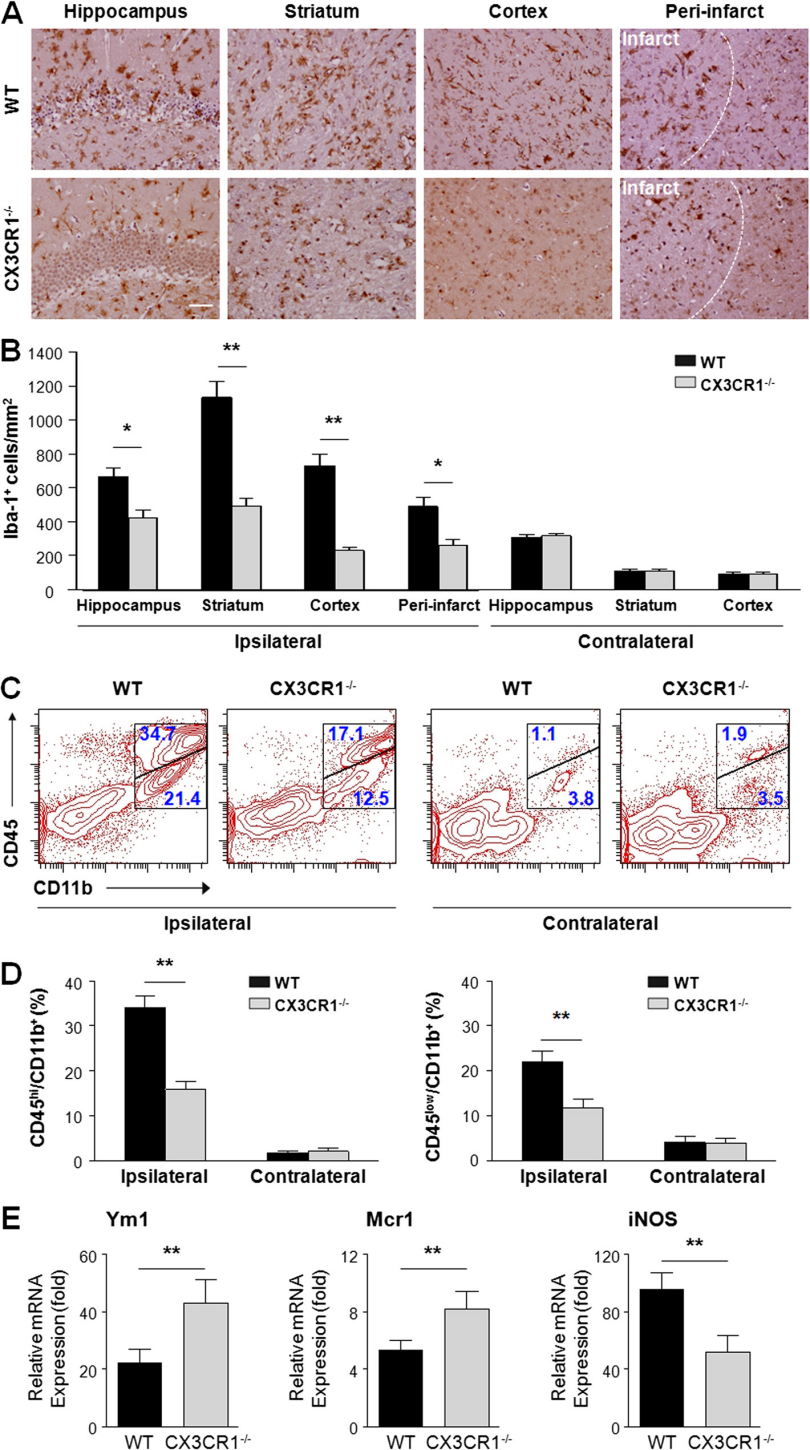
**CX3CR1 deficiency attenuates infiltration and leads to a different M1/M2 polarization pattern on microglia /macrophages in ipsilateral hemisphere of middle cerebral artery occlusion mice. (A)** Staining with anti-Iba-1 antibody in the ipsilateral hippocampus, striatum, cortex and peri-infarct zone of CX3CR1^-/-^ and wild-type (WT) mice 72 hours after middle cerebral artery occlusion (MCAO). **(B)** Quantification of Iba-1 positive cells. CX3CR1^-/-^ mice have fewer Iba-1 positive cells (macrophages/microglia) in the ipsilateral hippocampus, striatum, cortex and peri-infarct zone, while no differences are observed in these sites of the contralateral hemisphere. *P* < 0.0001 by two-way analysis of variance for genotype, localization and interaction. *P < 0.05, **P < 0.01 by Bonferroni *post-hoc* tests. *n* = 4 per group. Scale bars = 50 μm. **(C)** Flow cytometry analysis of CD45^hi^/CD11b^+^ and CD45^low^/CD11b^+^ cells isolated from the ipsilateral and contralateral hemispheres of CX3CR1^-/-^ and WT mice 72 hours after MCAO by gating on Ly6G^-^ events (Ly6G vs FSC). **(D)** Quantification of the number of events in the CD45^hi^/CD11b^+^/Ly6G^-^ (left) and CD45^low^/CD11b^+^ /Ly6G^–^ (right) gate. Statistical analysis shows CX3CR1^-/-^ mice have fewer CD45^hi^/CD11b^+^/Ly6G^-^ as well as CD45^low^/CD11b^+^ /Ly6G^–^ cells in the ipsilateral hemisphere. CD45^hi^/CD11b^+^/Ly6G^-^: *P* = 0.0001 for genotype, *P* < 0.0001 for localization and interaction by two-way analysis of variance. CD45^low^/CD11b^+^/Ly6G^-^: *P* = 0.0112 for genotype, *P* < 0.0001 for localization, and *P* = 0.0152 for interaction by two-way analysis of variance. **P < 0.01 by Bonferroni *post-hoc* tests. *n* = 4 per group. **(E)** Microglia/macrophage in ischemic hemisphere of CX3CR1^-/-^ brain polarize toward the M2 phenotype. Real-time reverse-transcription polymerase chain reaction was performed using total RNA extracted from sorted CD45^+^/CD11b^+^/Ly6G^-^ microglia/macrophage at 72 hours after MCAO. Data are expressed as fold change vs sham-operated controls. **P < 0.01 with Student’s *t*-test for Ym1, Mcr1, and iNOS, respectively. *n* = 4 per group.

Microglia activation plays an important role in the pathological progression after stroke, and is regulated by CX3CR1 [6,12]. To investigate the effects of CX3CR1 deficiency upon these processes, expression of microglia/macrophage activation marker Iba-1 was examined at different sites of the brain in WT and CX3CR1^-/-^ mice 72 hours following MCAO (Figure 3A). In the ipsilateral hemisphere of WT mice, the numbers of Iba-1 positive cells in hippocampus, striatum, cortex and peri-infract area (666.1 ± 50.0, 1132.8 ± 96.1, 730.9 ± 68.1, 489.4 ± 56.1/mm^2^) were significantly higher than those in CX3CR1^-/-^ mice (424.6 ± 43.1, 492.7 ± 42.3, 230.1 ± 20.1, 262.5 ± 29.8/mm^2^) (Figure 3B). In the contralateral hemisphere, the numbers of Iba-1 positive cells in the hippocampus, striatum and cortex were similar (307.9 ± 15.5 vs 316.0 ± 14.6, 110.2 ± 11.5 vs 111.8 ± 9.7, 94.0 ± 8.6 vs 92.4 ± 13.1, WT vs CX3CR1^-/-^) regardless of genotype (Figure 3B).

To further distinguish microglia from macrophages within the Iba-1 positive cell population, flow cytometry was used to delineate CD45^low^/CD11b^+^/Ly6G^-^ (microglia) and CD45^hi^/CD11b^+^/Ly6G^-^ (macrophage/activated microglia) sub-populations within ischemic lesions of WT and CX3CR1^-/-^ mice. As shown in Figure 3C,D, more macrophages/activated microglia infiltrated in the ischemia lesions in WT mice compared to CX3CR1^-/-^ mice (34.1 ± 2.5% vs 15.9 ± 1.8%). In addition, WT mice displayed more CD45^low^ microglia than CX3CR1^-/-^ mice (22.1 ± 2.3% vs 11.7 ± 2.1%, Figure 3C,D). Both cell populations were similar in WT and CX3CR1^-/-^ mice in the control (non-ischemic) contralateral hemisphere (Figure 3C,D).

Following ischemia, activated microglia/macrophage can potentially exert either a protective or detrimental effect, suggesting that these cells may acquire different phenotypes belonging to the classical (M1) or to the alternative (M2) active status. We found that activated M1-like Iba-1^+^ cells, which have shorter and thicker processes and bigger cell bodies, were visualized in the WT brain section, while ramified M2-like Iba-1^+^ cells were predominantly located in the CX3CR1^-/-^ brain (Figure 3A). To evaluate their M1/M2 polarization, we sorted and purified microglia/macrophages (CD45^+^/CD11b^+^/Ly6G^-^) from the ischemic hemisphere of CX3CR1^-/-^ and WT MCAO mice brain. Using real-time PCR, we found that the levels of tested M1- and M2-type genes in Fluorescence Activated Cell Sorter (FACS)-sorted microglia/macrophages from WT mice were increased starting from 1 day after MCAO and further elevated by 3 days post-MCAO (data not shown). Within CX3CR1^-/-^ microglia/macrophages, the M2-type genes (Ym1, Mcr1) were significantly increased, whereas the M1-type gene (iNOS) was notably decreased compared to WT microglia/macrophages when isolated at 72 hours after MCAO (Figure 3E). These results suggest that deficiency of CX3CR1 may facilitate the alternative activation (M2 state) of microglia/macrophages in stroke.

### Reduced proliferation of macrophages and microglia in ipsilateral hemisphere of CX3CR1^-/-^ mice after middle cerebral artery occlusion

**Figure 4:**
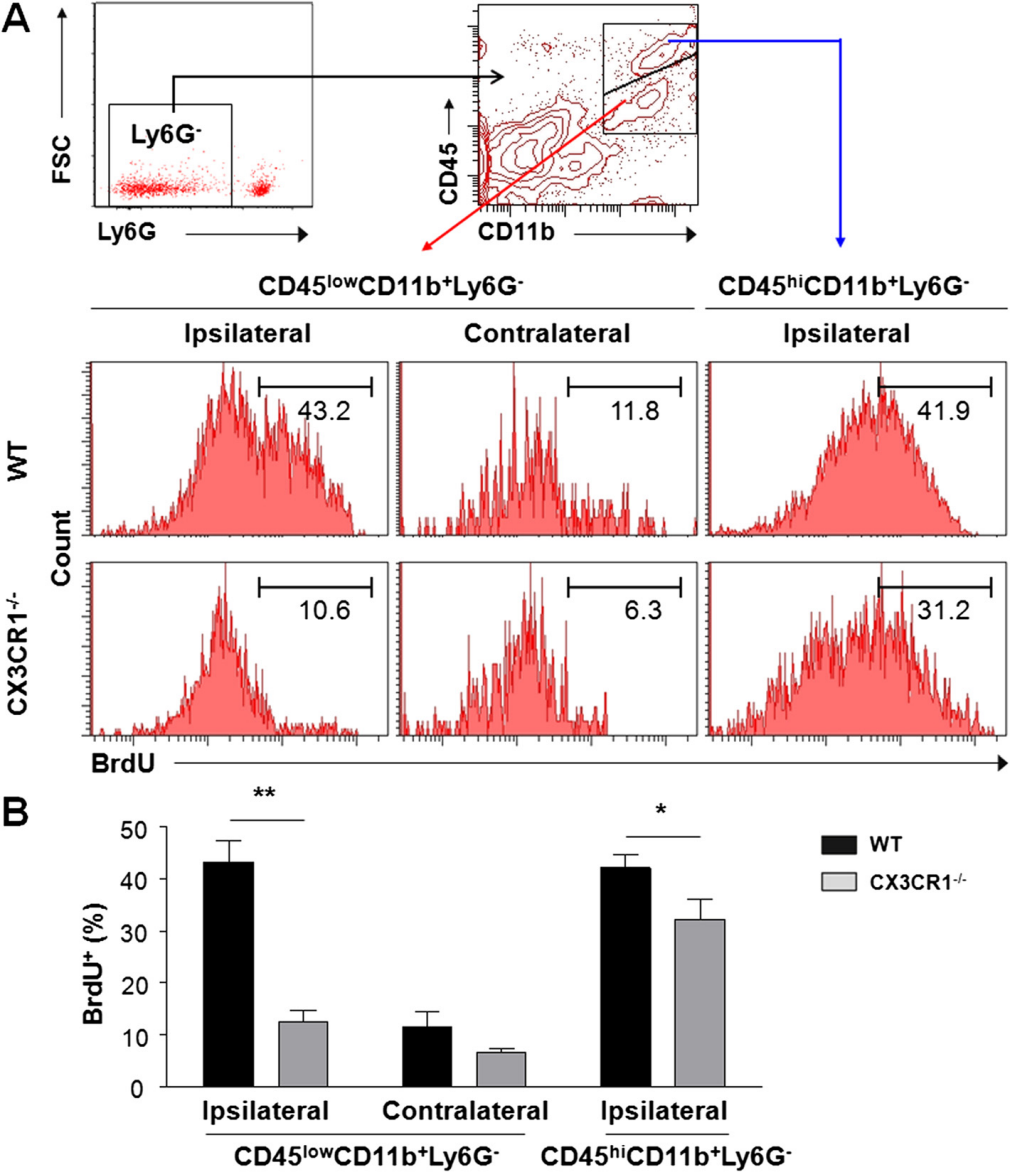
**Suppressed proliferation of CD45**^**low**^**/CD11b**^**+**^**/Ly6G**^**- **^**and CD45**^**hi**^**/CD11b**^**+**^**/Ly6G**^**- **^**cells in the ipsilateral hemisphere of CX3CR1**^**-/- **^**mice after middle cerebral artery occlusion. (A)** Flow cytometry analysis of proliferation of Ly6G^–^ gated CD45^low^/CD11b^+^ (microglia) and CD45^hi^/CD11b^+^ (macrophages/activated microglia) population with 5-bromo-2-deoxyuridine (BrdU) incorporation in the ipsilateral hemisphere of wild-type (WT) and CX3CR1^-/-^ mice 72 hours after middle cerebral artery occlusion (MCAO), compared to their contralateral (unlesioned) hemisphere controls. CD45^hi^/CD11b^+^/Ly6G^-^ cell proliferation was not shown in the contralateral due to their very low to undetectable presence. **(B)** Graph presents quantification of microglia/macrophage proliferation measured by flow cytometry. Data indicate a marked reduction in CD45^low^/CD11b^+^/Ly6G^-^ cell proliferation and a modest but significant reduction in CD45^hi^/CD11b^+^/Ly6G^-^ cell proliferation in the ipsilateral hemisphere of CX3CR1^-/-^ mice compared to WT mice 72 hours after MCAO. *P* < 0.0001 for genotype and localization, *P* = 0.0017 for interaction by two-way analysis of variance. *P < 0.05, **P < 0.01 by Bonferroni *post-hoc* tests. *n* = 4 per group.

To determine whether the decrease in CD11b^+^Ly6G^–^ cells observed in ischemic lesions of CX3CR1^-/-^ MCAO mice, in addition to decreased chemotaxis of monocytes, was due to suppressed expansion of microglia/macrophage, we assessed proliferation of CD11b^+^Ly6G^-^ cells. Proliferation analysis was performed on populations of CD45^low^/CD11b^+^/Ly6G^-^ cells (microglia), and CD45^hi^/CD11b^+^/Ly6G^-^ cells (macrophage/activated microglia) obtained from CX3CR1^-/-^ and WT mice injected with BrdU (Figure 4A). Quantitative analysis revealed a significant 3-fold increase in the number of CD45^low^/CD11b^+^/Ly6G^-^/BrdU^+^ cells in the ipsilateral relative to the contralateral hemisphere in WT mice 72 hours after stroke (Figure 4B). A significant reduction (71.1%) in the number of proliferating CD45^low^/CD11b^+^/Ly6G^-^ cells was observed in the ipsilateral hemisphere in CX3CR1^-/-^ compared to WT mice (WT, 43.3 ± 4.1%; CX3CR1^-/-^, 12.5 ± 2.2%, *P* < 0.01, Figure 4B). There were no significant differences in the numbers of CD45^low^/CD11b^+^/Ly6G^-^/BrdU^+^ cells in the contralateral hemisphere between the two experimental groups, although CX3CR1^-/-^ mice showed a lesser trend (WT, 11.5 ± 2.9%; CX3CR1^-/-^, 6.7 ± 0.7%, *P* > 0.05, Figure 4B). We also observed a modest but significant reduction (23.2%) in the number of proliferating CD45^hi^/CD11b^+^/Ly6G^-^ cells in the ipsilateral hemisphere in CX3CR1^-/-^ compared with WT mice (WT, 42.1 ± 2.7%; CX3CR1^-/-^, 32.3 ± 3.9%, *P* < 0.05). Collectively, these results indicate that CX3CR1 deficiency affects ischemic injury-induced proliferation of resident microglia and recruited macrophages.

### CX3CR1 deficiency attenuates reactive oxygen species generation in brain after middle cerebral artery occlusion

**Figure 5:**
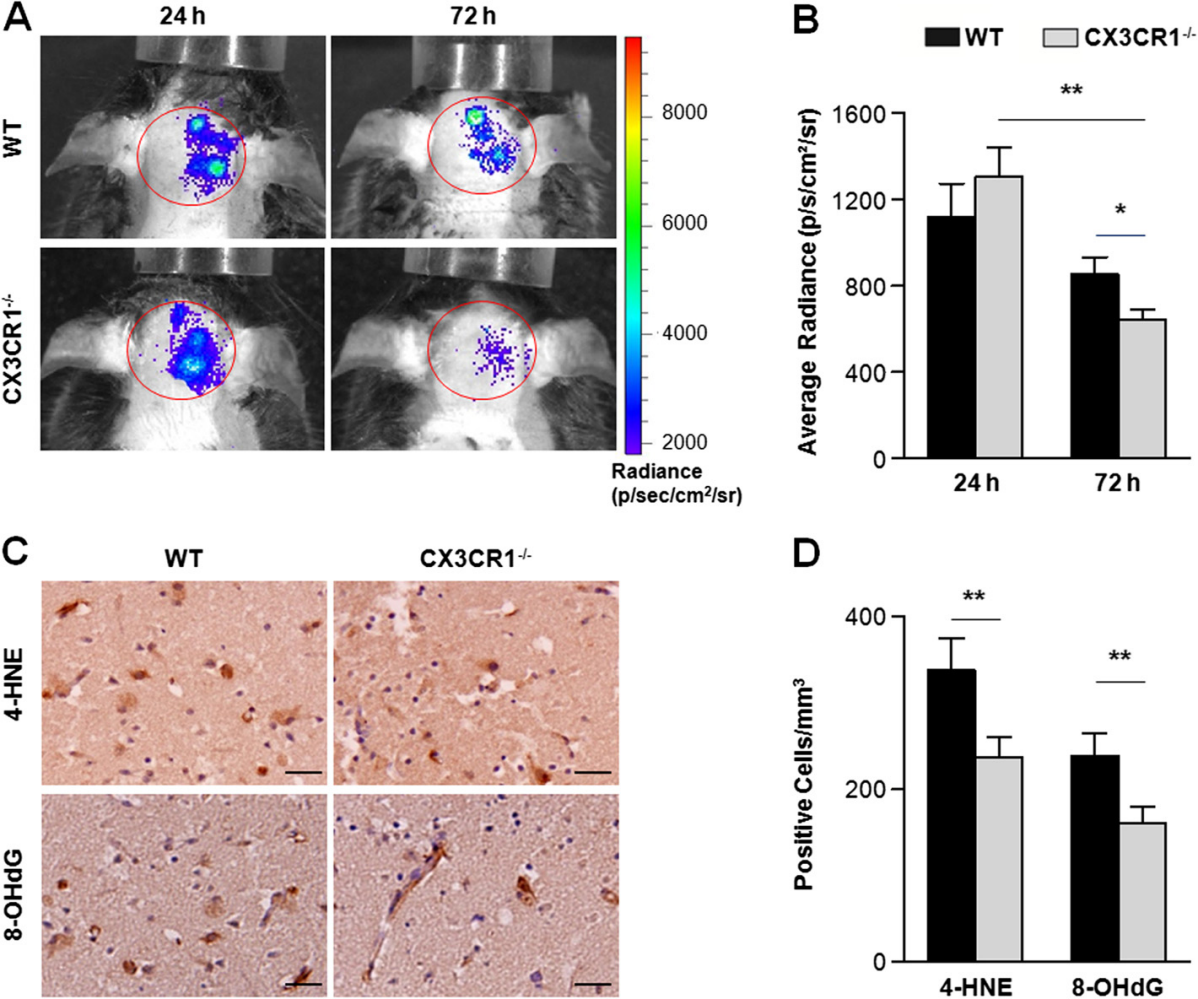
**CX3CR1 deficiency attenuates reactive oxygen species generation in brain after middle cerebral artery occlusion. (A)** Reactive oxygen species (ROS) were evaluated by Xenogen IVIS200 imager in wild-type (WT) and CX3CR1^-/-^ mice *in vivo*. **(B)** Quantification of ROS. No difference in ROS levels were observed between the two groups 24 hours after MCAO. ROS level decreased in CX3CR1^-/-^ mice 72 hours after MCAO compared to 24 hours (*P* < 0.0001 for genotype and time point, *P* = 0.0036 for interaction by two-way analysis of variance; **P < 0.01 by Bonferroni *post-hoc* tests) and is significantly less in CX3CR1^-/-^ mice relative to WT mice (*P < 0.05 by Bonferroni *post-hoc* tests). *n* = 6 per group. p/s/cm^2^/sr, photons per second per centimeter squared per steradian. **(C)** Immunohistochemistry for 4-hydroxy-2-nonenal (4-HNE) and 8-hydroxy-2-deoxyguanosine (8-OHdG) in the ischemic lesion 24 and 72 hours after MCAO. **(D)** Reduction of the number of stained cells in the CX3CR1^-/-^ mice compared with WT mice. *P* < 0.0001 for genotype, *P* = 0.0003 for oxidative marker, and *P* = 0.9827 for interaction by two-way analysis of variance. **P < 0.01 by Bonferroni *post-hoc* tests. *n* = 5 per group scale bars: 50 μm.

ROS, generated as by-products of cellular metabolism, have long been known to be a component of the inflammatory response after ischemia. To investigate whether CX3CR1 deficiency had effects upon ROS production, we assessed ROS levels in live mice using the Xenogen IVIS200 imager at 24 and 72 hours after MCAO. The chemiluminescence detection of ROS was performed in the brains, specifically in the ipsilateral hemisphere (Figure 5A). The mean chemiluminescence intensity of brain in WT mice was 1,090.3 ± 127.4 p/s/cm^2^/sr (photons per second per centimeter squared per steradian) at 24 hours and 850.9 ± 81.7 p/s/cm^2^/sr at 72 hours. Within CX3CR1^-/-^ mice, mean chemiluminescence intensity values were 1,350.9 ± 118.9 p/s/cm^2^/sr at 24 hours and 641.7 ± 47.6 p/s/cm^2^/sr at 72 hours. At 24 hours post-ischemia, no differences in ROS levels were observed between the two groups (*P* > 0.05). At 72 hours post-ischemia, the ROS levels decreased significantly in CX3CR1^-/-^ mice compared to 24 hours (*P* < 0.01), while no change was observed in WT mice (*P* > 0.05) (Figure 5B). In addition, the oxidative impairment of neurons was immunohistochemically assessed by stain for lipid peroxidation with 4-HNE and damaged DNA with 8-OHdG (Figure 5C). The number of stained cells in the CX3CR1^-/-^ mice was notably less compared with WT mice (*P* < 0.01) (Figure 5D).

### CX3CR1 deficiency impairs inflammatory signaling in microglia and macrophage in ischemic brain

**Figure 6:**
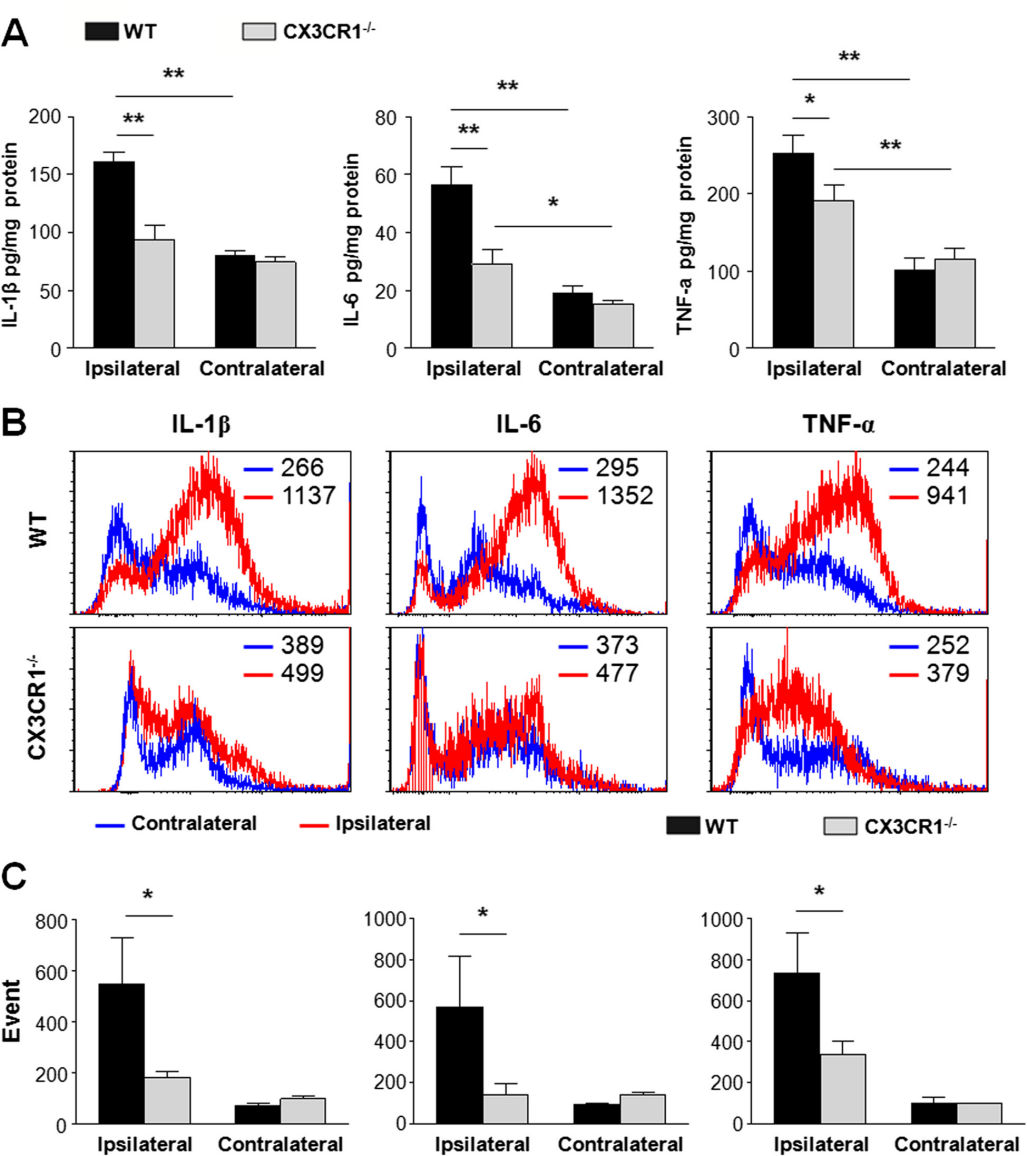
**CX3CR1 deficiency impairs inflammatory signaling in microglia and macrophage in ischemic brain. (A)** The amounts of IL-1β, IL-6, and TNF-α in brain homogenates from wild-type (WT) and CX3CR1^-/-^ mice 72 hours after middle cerebral artery occlusion (MCAO) were measured with ELISA. IL-1β: *P* = 0.0018 for genotype, *P* = 0.0002 for localization, and *P* = 0.0052 for interaction by two-way analysis of variance; IL-6: *P* = 0.0010 for genotype, *P* < 0.0001 for localization, and *P* = 0.0058 for interaction by two-way analysis of variance; TNF-α: *P* < 0.0001 for genotype, *P* = 0.0240 for localization, and *P* = 0.0063 for interaction by two-way analysis of variance. *P < 0.05, **P < 0.01 by Bonferroni *post-hoc* tests. *n* = 4 per group. **(B)** IL-1β, IL-6, and TNF-α expression were analyzed by flow cytometry within the CD11b^+^Ly6G^–^ gate. Representative histograms show IL-1β, IL-6, and TNF-α expression in the contralateral (blue) and ipsilateral (red) hemispheres of CX3CR1^-/-^ and WT mice at 72 hours after MCAO. Mean fluorescent intensity is indicated within each representative histogram. **(C)** The number of IL-1β, IL-6, and TNF-α-producing CD11b^+^Ly6G^–^ cells was quantified from ischemic brain of CX3CR1^-/-^ and WT mice at 72 hours after MCAO with flowcytometry. IL-1β^+^/CD11b^+^/Ly6G^–^: *P* = 0.0030 for genotype, *P* = 0.0002 for localization, and *P* = 0.0052 for interaction by two-way analysis of variance; IL-6^+^/CD11b^+^/Ly6G^–^: *P* = 0.0030 for genotype, *P* = 0.0002 for localization, and *P* = 0.0052 for interaction by two-way analysis of variance; TNF-α^+^/CD11b^+^/Ly6G^–^: *P* = 0.0386 for genotype, *P* = 0.0332 for localization, and *P* = 0.0173 for interaction by two-way analysis of variance. *P < 0.05 by Bonferroni *post-hoc* tests. *n* = 4 per group.

To determine whether CX3CR1 deficiency is associated with changes in the expression of inflammatory mediators produced by activated macrophages and microglia, ELISA was used to screen injured brain homogenates for differences in cytokine production. Consistent with previous reports [25-27], expression of a subset of inflammatory cytokines (IL-1β, IL-6, and TNF-α) was increased after MCAO regardless of genotype. In WT mice, the amounts of IL-1β, IL-6, and TNF-α were 160.9 ± 8.4, 56.5 ± 6.1, 253.0 ± 22.9 pg/mg in the ipsilateral hemisphere and 80.7 ± 4.1, 19.2 ± 2.2, 101.9 ± 15.3 pg/mg in the contralateral hemisphere, respectively. In CX3CR1^-/-^ mice, the amounts of IL-1β, IL-6, and TNF-α were 94.2 ± 11.9, 29.0 ± 5.1, 191.9 ± 19.8 pg/mg in the ipsilateral hemisphere and 74.5 ± 5.1, 15.5 ± 1.1, 116.5 ± 13.7 pg/mg in the contralateral hemisphere, respectively. Notably, post-injury expressions of these cytokines were markedly reduced in CX3CR1^-/-^ mice in the ipsilateral hemisphere, while no difference was observed in the contralateral hemisphere (Figure 6A).

To determine whether deficient CX3CR1 signaling in CNS microglia/macrophages could account for these cytokine expression changes, a series of controlled *ex vivo* flow cytometry assays were performed. Microglia and macrophages were isolated from ischemic brains of WT and CX3CR1^-/-^ mice, followed by quantification of IL-1β^+^/CD11b^+^/Ly6G^–^, IL-6^+^/CD11b^+^/Ly6G^–^, and TNF-α^+^/CD11b^+^/Ly6G^–^ cells using flow cytometry. Using this approach, an increased expression of IL-1β, IL-6, and TNF-α in CD11b^+^Ly6G^–^ cells (fluorescent intensity in Figure 6B) as well as the numbers of cytokine-expressing CD11b^+^Ly6G^–^ cells (event quantification in Figure 6C) were detected 72 hours after MCAO in the ischemic lesions in WT mice (ipsilateral vs contralateral). This response was noticeably absent from the injured brain of CX3CR1^-/-^ mice (Figure 6B,C). In the ipsilateral hemisphere, CX3CR1^-/-^ mice displayed significant reduction in expression of IL-1β, IL-6, and TNF-α in CD11b^+^Ly6G^–^ cells and cytokine-expressing CD11b^+^Ly6G^–^ cell numbers (*P* < 0.05 vs WT, Figure 6B,C).

## Discussion

The work presented here provides important *in vivo* evidence for the role of the CX3CL1/CX3CR1 signaling pathway in the activation and neurotoxicity of microglia/ macrophage in cerebral ischemia. Using CX3CR1^-/-^ mice, in which the CX3CR1 gene is substituted with the gene for the GFP on both alleles (CX3CR1^GFP/GFP^), and a murine model of transient brain ischemia, we find that CX3CR1 deficiency resulted in a decrease in the size of ischemic lesion, a decrease in the number of apoptotic cells (predominantly neurons), marked deregulation of post-ischemic brain inflammatory responses (including ROS and pro-inflammatory cytokines), a significant decrease in proliferation of microglia and infiltrating macrophages in the ischemic lesion, and a marked decrease in the levels of IL-1β/IL-6/TNF-α expressed by microglia/macrophage in ischemic brain. Collectively, these effects due to CX3CR1 deficiency correlate with improved neurological function following MCAO and suggest that blockade of CX3CR1/CX3CL1 signaling may provide neuroprotection against ischemic injury.

Absence of fractalkine receptor in CX3CR1^-/-^ mice has been previously reported to be protective in ischemia. Critically, significantly smaller (56%) infarcts and blood–brain barrier damage have been observed in CX3CR1^-/-^ animals compared with CX3CR1^+/-^ and WT mice 72 hours after a 60-minute MCAO [15]. CX3CR1 deficiency has also been shown to induce protection from a 30-minute MCAO starting as early as 24 hours [17]. In an experimental model of spinal cord injury, CX3CR1^-/-^ mice demonstrate neuroprotection and functional recovery 5 days post-injury [16]. In agreement with these previous studies on the role of the CX3CL1/CX3CR1 pathway in acute CNS injury, our work finds that mice lacking CX3CR1 are protected from ischemic injury 72 hours after a 90-minute MCAO. Interestingly, we find prevention of exacerbation of ischemic lesion was accompanied with reduced cleaved Caspase 3 positive neurons in the peri-infarct area at 72 hours after initial stroke in CX3CR1^-/-^ mice compared to WT mice, inconsistent with a previous report [15]. While these observed differences could be attributed to the differences in the experimental designs, the outcomes between our study and others are identical. Further, it may also imply that the effects of CX3CL1/CX3CR1 signaling may be dependent upon the context of the specific disease state.

CX3CR1 deficiency has been suggested to attenuate tissue damage and improve recovery of function by reducing the recruitment and/or the activation of microglia and macrophages [15,28-30]. We tested this hypothesis by isolating mononuclear cells from the injured brain of WT and CX3CR1^-/-^ mice, followed by quantifying CD45^+^/CD11b^+^ cells by flow cytometry. We find a significant decrease in CD45^+^/CD11b^+^/Ly6G^–^ microglia/macrophage in the ipsilateral hemisphere of CX3CR1^-/-^ mice vs WT mice, which was confirmed by immunohistochemical staining with Iba-1 in brain sections. To determine whether this was a result of suppressed microglia proliferation or decreased monocyte recruitment, the relative expression of CD45 has been used to differentiate resident microglia (CD45^low^) from recruited monocytes (CD45^hi^) [31]. CD45^low^/CD11b^+^/Ly6G^–^ cells (microglia) predominate in the contralateral side of both genotypes. However, by 72 hours post-injury, accumulation of both CD45^low^ (microglia) and CD45^hi^ (macrophage/activated microglia) CD11b^+^/Ly6G^–^ cells dramatically decreased in the ischemic hemisphere of CX3CR1^-/-^ mice. Moreover, BrdU assays show that the proliferation of microglia (CD45^low^) is significantly impaired in CX3CR1^-/-^ MCAO mice. The proliferation of CD45^hi^ cells (including activated microglia) was also suppressed; however, not to the extent which could fully compensate for the decreased number of CD45^hi^ cells seen in CX3CR1^-/-^ MCAO mice compared to WT MCAO mice. We therefore speculate that the migration of monocyte-derived macrophages from the periphery was inhibited. These data together indicate that both microglia/macrophage proliferation and macrophage recruitment are attenuated by CX3CR1 deficiency within the MCAO model. Our results coincide with a previous study that demonstrated decreased leukocytes infiltration as a possible mechanism for the development of smaller infarcts in CX3CR1^-/-^ mice after a 60-minute MCAO [15]. Conversely, more CD45^hi^ cells were found accumulated in the injured spinal cords of CX3CR1^-/-^ mice. Although the role of this cell population is not clear (rather, this study revealed a distinct monocyte subset), CD11b^+^/Ly6C^lo^/iNOS^+^ macrophages were associated with reduced neuropathology and enhanced functional recovery [16]. Indeed, it has long been thought that macrophage (microglia) are not a uniform cell population, such that specific environmental signals, including effector molecules, timing of activation, and degree of injury, may induce their different polarization states [32,33]. Therefore, phenotypically distinct monocyte populations appear to be associated with tissue pathology and repair in ischemia and, thus, warrant further study [16].

Previous reports indicated a marked increase in proinflammatory cytokine levels that peak 12 to 24 hours after ischemic injury [34]. Our results obtained from the WT mice are in agreement with these temporal dynamics of cytokines. As expected, an initial increase of TNF-α, IL-1β, and IL-6 was detected in the ipsilateral hemisphere after MCAO. However, contrary to the levels of proinflammatory cytokines observed in control samples, we show that the ablation of proliferating microglia cells and recruitment of monocytes, associated with CX3CR1 deficiency, resulted in a significant decrease in the levels of IL-1β, TNF-α, and IL-6, suggesting an important temporal deregulation of the proinflammatory cytokine response. Similar patterns of mRNA expression and temporal deregulations were observed in the NF-κB signaling pathway, a general marker of inflammatory response in cerebral ischemia [16]. Furthermore, using flow cytometry, we confirmed that the trends of proinflammatory cytokines in the ipsilateral hemisphere of the MCAO brain in CX3CR1^-/-^ mice are consistent with the levels secreted by CX3CR1^-/-^ microglia/macrophage isolated from MCAO brains. Therefore, it appears that microglia/macrophage may play an important role in the regulation/modulation of the proinflammatory responses in cerebral ischemia, and their functions are attenuated by CX3CR1 deficiency.

Variations of cell morphology or cell displacement are related to the specific activity of microglia/macrophage [32]. Following ischemia, activated microglia/macrophage can potentially exert either a protective or detrimental effect, suggesting that these cells may acquire different phenotypes belonging to the classical (M1) or to the alternative (M2) active status [35]. M1 activation is generally referred to as the pro-inflammatory and cytotoxic phenotype, characterized by nitric oxide, ROS, and proinflammatory cytokine production, while the M2 phenotype is an alternative activation state, associated with a fine tuning of inflammation, scavenging of debris, promotion of angiogenesis, tissue remodeling and repair [17,36]. Fumagalli and colleagues [17] reported that, in CX3CR1^-/-^ mice, protection from ischemia at early time points after injury is associated with a protective inflammatory milieu, characterized by the promotion of M2 polarization markers. We found that activated M1-like Iba-1^+^ cells, which have shorter and thicker processes and bigger cell bodies, were visualized in the WT brain section, while ramified M2-like Iba-1^+^ cells were predominantly located in the CX3CR1^-/-^ brain (Figure 3A) with increased expression of M2 markers (Figure 3E), implying that suppressed activation of microglia/macrophage by CX3CR1 deficiency may be responsible for the neuroprotective effects in the CX3CR1^-/-^ mice.

## Conclusion

In summary, using a murine model of transient focal ischemia, we explored the consequences of CX3CR1 absence on microglia/macrophage proliferation/recruitment and on their activation after brain ischemia. When CX3CR1 signaling is abolished, neurological recovery is improved and neural pathology is reduced. Our data show that loss of CX3CR1 signaling attenuates the proliferation and inflammatory capacity of microglia/macrophages. Moreover, monocytes recruitment and/or the differentiation of newly recruited monocyte subsets are altered. Collectively, these data identify CX3CR1 as a putative therapeutic target for selectively reducing neurodestructive inflammatory cascades after stroke.

## Abbreviations

4-HNE: 4-hydroxy-2-nonenal; 8-OHdG: 8-hydroxy-2-deoxyguanosine; ADC: apparent diffusion coefficient; BrdU: 5-bromo-2-deoxyuridine; CBF: cerebral blood flow; CNS: central nervous system; CX3CL1: chemokine (C-X3-C motif) ligand 1; CX3CR1: CX3C chemokine receptor 1; CX3CR1-/-: CX3CR1-deficient; ELISA: enzyme-linked immunosorbent assay; FACS: Fluorescence Activated Cell Sorter; GFP: green fluorescent protein; IL: interleukin; MCAO: middle cerebral artery occlusion; MRI: magnetic resonance imaging; PBS: phosphate-buffered saline; PCR: polymerase chain reaction; ROS: reactive oxygen species; TNF: tumor necrosis factor; TTC: 2,3,5-triphenyltetrazolium chloride; WT: wild-type.

## Competing interests

The authors declare that they have no competing interests.

## Authors’ contribution

ZT and YG designed the experiments. ZT, YG, QL, J-XY and QL performed the experiments. ZT and YG analyzed the data and wrote the paper. JS and F-DS supervised the experimental work. F-DS obtained the funding and resources. All authors read and approved the final manuscript.

## Supplementary Material

Additional file 1 Supplemental Methods and Figures. Click here for file
