# Supplementary material for: CX3CR1 deficiency suppresses activation and neurotoxicity of microglia/macrophage in experimental ischemic stroke
Source: J Neuroinflammation. 2014 Feb 3;11:26. doi: 10.1186/1742-2094-11-26 (PMC3942808; doi:10.1186/1742-2094-11-26)
Supplement: Additional file 1 — Supplemental Methods and Figures. [file 1742-2094-11-26-S1.doc]

**SUPPLEMENTARY INFORMATION**

**CX3CR1 Deficiency Suppresses Activation and Neurotoxicity of Microglia/Macrophage in Experimental Ischemic Stroke**

Zhiwei Tang^1,2,†^, Yan Gan^1,†^, Qiang Liu^1,3^, Jun-xiang Yin^1^, Qingwei Liu^1^, Jiong Shi^1^, Fu-Dong Shi^1,3^,*

^1^ Department of Neurology and the BNI-ASU Center for Preclinical Imaging, Barrow Neurological Institute, St. Joseph’s Hospital and Medical Center, Phoenix, Arizona 85013, USA

^2^ Department of Neurosurgery, the First Affiliated Hospital of Kunming Medical University, Kunming 650031, China

^3^ Department of Neurology, Tianjin Neurological Institute, Tianjin Medical University General Hospital, Tianjin 300052, China

^†^ These authors contributed equally to the paper.

* Corresponding author. Department of Neurology, Barrow Neurological Institute, St. Joseph’s Hospital and Medical Center, 350 W Thomas Rd, Phoenix, Arizona 85013, USA. Fax: +1 602 406 8765. E-mail address: fshi@dignityhealth.org

**Supplemental Methods**

**Transient Middle Cerebral Artery Occlusion (MCAO) and Reperfusion**

The mice were exposed to transient MCAO for 90 minutes, as described previously . Briefly, under Ketamine/Xylazine anesthesia (80 mg/kg Ketamine and 10mg/kg Xylazine, injected intraperitoneally), a 6-0 surgical nylon monofilament with rounded tip was introduced into the right internal carotid artery through the external carotid stump, then advanced 10-11 mm past the carotid bifurcation until a slight resistance was felt. At this point, the intraluminal filament blocked the origin of the middle cerebral artery. The filament was withdrawn 90 minutes after occlusion and reperfusion was induced. Body temperature of mice was maintained at 36.5 ± 0.5°C during surgery. Mice were intraperitoneally injected with 1 ml of sterile saline daily.

**2,3,5-Tripenyltetrazolium Chloride (TTC) Staining**

In order to confirm the infarction volume determined by T2-weighted MRI images, TTC staining was performed immediately after MRI scanning. Briefly, animals were anaesthetized with isoflurane and the brains were removed rapidly and frozen at -20°C for 5 minutes. Coronal slices were made at 1–2 mm from the frontal tips, and sections were immersed in 2% TTC (Sigma) at 37°C for 20 minutes. The presence of infarction was determined by the area that was not stained with TTC.

**CBF, ADC, and T2-Weighted Images by Rodent Magnetic Resonance Imaging (MRI)**

MR studies were carried out on a 7T small animal MRI, 30-cm horizontal-bore magnet, and BioSpec Avance III spectrometer (Bruker Daltonics Inc, Fremont, CA) with a 116-mm high power gradient set (600 mT/m) and a 72-mm whole-body mouse transmit/surface receive coil configuration. Single slice cerebral blood flow (CBF) images were acquired using a Continuous Arterial Spin Labeling (CASL) sequence with parameters: TR = 3000 ms, TE = 6.95 ms, segments = 4, slice thickness = 1.5mm, field of View (FOV) 2.0 cm × 2.0 cm, matrix 64 × 64, total scan time 20 min, to evaluate the successful induction of ischemia and reperfusion. Diffusion tensor images were acquired for calculating the apparent diffusion coefficient (ADC) maps 30 min after MCAO by Multiple Segments EPI sequences with the following parameters: TR = 5000 ms, TE = 30 ms, 0.5 mm slice thickness without slice gap, field of View (FOV) 2.56 cm × 2.56 cm, matrix 128 × 128, 18 slices, total scan time 2 min 20 sec, 6 direction of B were applied, with B = 100, 200, 400, 600, 800, 1000s/mm^2^ along each direction. T2-weighted images were acquired to locate the volume of interest and assess the infarct lesions, using a Rapid Acquisition with Refocused Echoes (RARE) sequence with parameters: TR = 3000 ms, effective TE = 60 ms, RARE factor = 8. 20 slices were acquired with thickness = 0.5mm, field of View (FOV) 2.56 cm × 2.56 cm, matrix 128 × 128, total scan time 3 min 12 sec. Image data were analyzed using the MED × 3.4.3 software package (Medical Numerics Inc, Germantown, MA) on a LINUX workstation.

**Flow Cytometry**

Cells isolated from brain tissue were stained in two experimental sets. Briefly, cells were first incubated in blocking solution (anti-CD16/32 antibody, BD Biosciences). Samples were then split into two sets for staining. Samples from one set were stained with the following antibodies: APC-conjugated anti-CD45 and PE-Cy7-conjugated anti-CD11b for 30 min. After washing, the samples were then fixed, permeabilized with BD Cytofix/Cytoperm and BD Cytoperm Plus for both cell membrane and nuclear membrane, and DNase (BD Biosciences) treated. Afterward, cells were incubated in normal mouse serum (eBioscience) for 15 min, and then stained with PerCP-Cy5.5-conjugated anti-BrdU for 20 minutes. In a separate set of experiments, cells were stained with the following antibodies: PerCP-Cy5.5-conjugated anti-CD45 and PE-Cy7-conjugated anti-CD11b for 30 min. After fixation and permeabilization with BD Cytofix/Cytoperm for cell membrane, cells were incubated with PE-conjugated anti-IL-1β, V450-conjugated anti-IL-6, and APC-conjugated anti-TNF-α for 30 minutes. To avoid the interference of GFP expressed on CX3CR1^-/-^ microglia/macrophage, fluors such as FITC and Alexafluor 488 that excite in the same spectrum at the same filter sets as GFP were excluded.

After all staining was completed, cells were washed and analyzed using a FACSAria Ι flow cytometer (BD Biosciences). Data were processed using FACSDiva software or FCS Express 4 software (BD Biosciences).

**Supplemental Figures and Figure Legends**

**
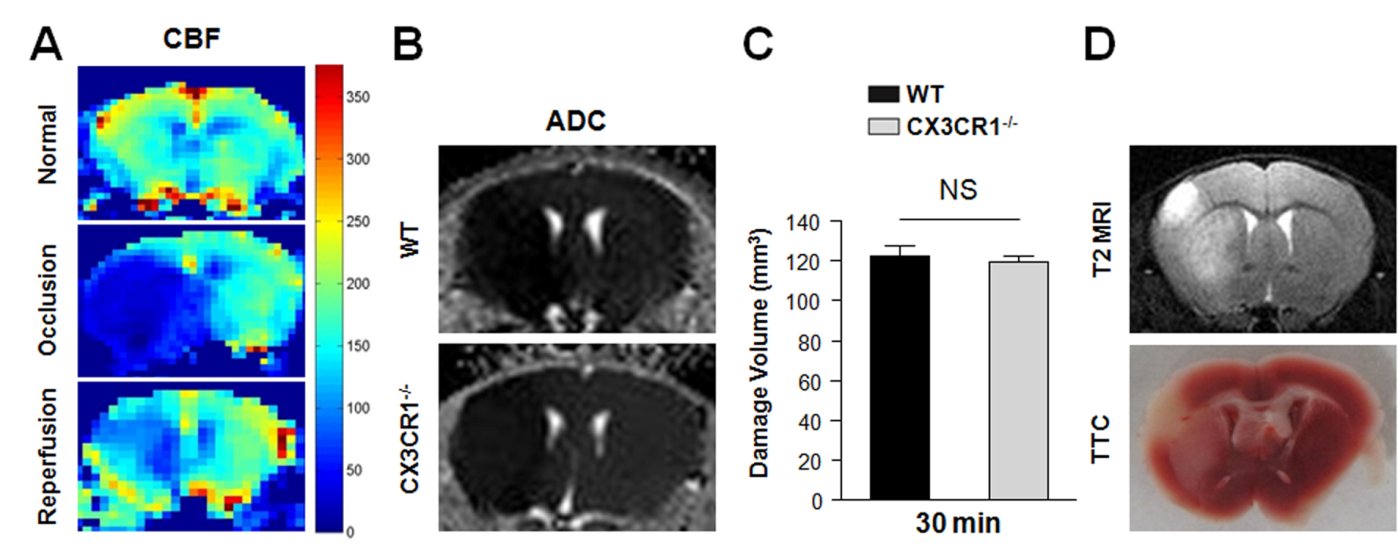
**

**Supplemental Figure S1. CBF, ADC, T2-Weighted MRI, and TTC images after transient middle cerebral artery occlusion (MCAO) and reperfusion.**

(**A**) Single slice cerebral blood flow (CBF) via MRI. The blood flow of right middle cerebral artery territory (normal, upper panel) decreased dramatically 1 hour after occlusion (middle panel), and increased successfully 24 hours after reperfusion (lower panel). Unit: ml/100g/min. (**B**) Damage volume was assessed by apparent diffusion coefficient (ADC) images. (**C**) Quantification of ADC maps shows that the damage volume 30 minutes after occlusion is similar between WT and CX3CR1^-/-^ mice. Students’ t-test. NS, not significant. *n* = 6 per group. (**D**) The infarction volume shown in T2-weighted MRI was confirmed by TTC staining.

**Supplemental References**

**1.** Chen J, Zacharek A, Zhang C, Jiang H, Li Y, Roberts C, Lu M, Kapke A, Chopp M: **Endothelial nitric oxide synthase regulates brain-derived neurotrophic factor expression and neurogenesis after stroke in mice.** *The Journal of neuroscience : the official journal of the Society for Neuroscience* 2005, **25:**2366-2375.

2. Liesz A, Suri-Payer E, Veltkamp C, Doerr H, Sommer C, Rivest S, Giese T, Veltkamp R: **Regulatory T cells are key cerebroprotective immunomodulators in acute experimental stroke.** *Nature medicine* 2009, **15:**192-199.

3. Tureyen K, Vemuganti R, Sailor KA, Dempsey RJ: **Infarct volume quantification in mouse focal cerebral ischemia: a comparison of triphenyltetrazolium chloride and cresyl violet staining techniques.** *Journal of neuroscience methods* 2004, **139:**203-207.
